# Prediction-Driven Decision Support for Patients With Mild Stroke: A Model Based on Machine Learning Algorithms

**DOI:** 10.3389/fneur.2021.761092

**Published:** 2021-12-23

**Authors:** Xinping Lin, Shiteng Lin, XiaoLi Cui, Daizun Zou, FuPing Jiang, JunShan Zhou, NiHong Chen, Zhihong Zhao, Juan Zhang, Jianjun Zou

**Affiliations:** ^1^School of Basic Medicine and Clinical Pharmacy, China Pharmaceutical University, Nanjing, China; ^2^Department of Clinical Pharmacology, Nanjing First Hospital, Nanjing Medical University, Nanjing, China; ^3^Department of Neurology, Nanjing Yuhua Hospital, Yuhua Branch of Nanjing First Hospital, Nanjing Medical University, Nanjing, China; ^4^Department of Geriatrics, Nanjing First Hospital, Nanjing Medical University, Nanjing, China; ^5^Department of Neurology, Nanjing First Hospital, Nanjing Medical University, Nanjing, China; ^6^Department of Neurology, The First Affiliated Hospital (People's Hospital of Hunan Province), Hunan Normal University, Changsha, China; ^7^Department of Clinical Pharmacology, Nanjing First Hospital, China Pharmaceutical University, Nanjing, China

**Keywords:** mild stroke, machine learning, post-stroke disability, decision support tool, predictive model

## Abstract

**Background and Purpose:** Treatment for mild stroke remains an open question. We aim to develop a decision support tool based on machine learning (ML) algorithms, called DAMS (Disability After Mild Stroke), to identify mild stroke patients who would be at high risk of post-stroke disability (PSD) if they only received medical therapy and, more importantly, to aid neurologists in making individual clinical decisions in emergency contexts.

**Methods:** Ischemic stroke patients were prospectively recorded in the National Advanced Stroke Center of Nanjing First Hospital (China) between July 2016 and September 2020. The exclusion criteria were patients who received thrombolytic therapy, age <18 years, lack of 3-month modified Rankin Scale (mRS), disabled before the index stroke, with an admission National Institute of Health stroke scale (NIHSS) > 5. The primary outcome was PSD, corresponding to 3-month mRS ≥ 2. We developed five ML models and assessed the area under curve (AUC) of receiver operating characteristic, calibration curve, and decision curve analysis. The optimal ML model was selected to be DAMS. In addition, SHapley Additive exPlanations (SHAP) approach was introduced to rank the feature importance. Finally, rapid-DAMS (R-DAMS) was constructed for a more urgent situation based on DAMS.

**Results:** A total of 1,905 mild stroke patients were enrolled in this study, and patients with PSD accounted for 23.4% (447). There was no difference in AUCs between the five models (ranged from 0.691 to 0.823). Although there was similar discriminative performance between ML models, the support vector machine model exhibited higher net benefit and better calibration (Brier score, 0.159, calibration slope, 0.935, calibration intercept, 0.035). Therefore, this model was selected for DAMS. In addition, SHAP approach showed that the most crucial feature was NIHSS on admission. Finally, R-DAMS was constructed and there was similar discriminative performance between R-DAMS and DAMS, but the former performed worse on calibration.

**Conclusions:** DAMS and R-DAMS, as prediction-driven decision support tools, were designed to aid clinical decision-making for mild stroke patients in emergency contexts. In addition, even within a narrow range of baseline scores, NIHSS on admission is the strongest feature that contributed to the prediction.

## Introduction

Around half of patients with ischemic stroke have mild neurological symptoms (1), usually with the expectation that such patients will come back to their pre-stroke activities regardless of the treatment. However, over one-third of mild stroke patients present with some degree of post-stroke disability (PSD) (2–4), which may be the result of inadequate acute treatments, early stroke recurrence, serious complications, or other reasons (1, 5). For the acute treatment of mild stroke patients, the guidelines from the American Heart Association/American Stroke Association (AHA/ASA) (6) distinguish disabling from non-disabling stroke and recommend intravenous (IV) alteplase only for the former. Nonetheless, the more certain, but not definitive, concept of “disabling stroke” is subjective and requires interpretation by individual neurologists. On the other hand, there is a trade-off between the benefits of IV alteplase and the risk of symptomatic intracranial hemorrhage (sICH). Therefore, decisions on how to treat mild stroke patients should be made on an individual basis.

3-month modified Rankin Scale (mRS), a valuable instrument for testing therapeutic interventions (7, 8), was used to assess the levels of PSD (5, 8). For mild stroke patients who only received medical therapy but had PSD, such therapy is not enough. Therefore, mild stroke patients who would be at high risk of PSD if they only received medical therapy should be early identified in emergency contexts, and some aggressive treatments, such as IV alteplase or close monitoring preventing worsening, should be taken in time. Unexpectedly, neurologists' overall accuracy for identifying those patients was staggeringly low (16.9%) (9). Each day that such a problem continues to exist means that uncounted mild stroke patients are being left with preventable disability.

However, none of the previously published risk models which were developed to predict the function outcome after stroke are fit to solve this problem. For example, the Totaled Health Risks in Vascular Events (THRIVE) score and the Houston Intra-Arterial Therapy (HIAT) score assign 0 points for National Institute of Health stroke scale (NIHSS) ≤ 5, losing the predictive power of NIHSS in mild stroke patients (10, 11). NIHSS on admission has been proven to be a strong predictor of PSD (5). Thus, despite convenient clinical applicability, these models cannot accurately identify mild stroke patients at high risk of PSD. Such models remain inadequate.

With the increased clinical data gathered for each patient, modern medical decision-making demands accurate, novel, and prediction-driven decision support. Machine learning (ML) algorithm, as a burgeoning statistical approach, is well-suited for that mission. Numerous studies with a considerable number of patients have shown great potential for ML approaches to predict recurrence (12), swallowing recovery (13), or aphasia (14) in patients with stroke. However, a model based on ML algorithms, focusing on the more debatable area of treating MS, has not yet been established.

Here, our goal was to develop and validate a prediction-driven decision support tool based on ML algorithms, called DAMS (Disability After Mild Stroke), to early identify mild stroke patients who would be at high risk of PSD if they only received medical therapy, and more importantly, to assist neurologists to make individual clinical decisions for mild stroke patients.

## Materials and Methods

### Study Population

The study population involved the sequential ischemic stroke patients within 12 h of symptoms onset recorded in the National Advanced Stroke Center of Nanjing First Hospital (China) between July 2016 and September 2020. The exclusion criteria were patients who received thrombolytic therapy, age <18 years, lack of 3-month mRS, who were disabled before the stroke (premorbid mRS score ≥ 2), with an admission NIHSS > 5. The primary outcome was PSD, corresponding to 3-month mRS ≥ 2.

Based on the Helsinki declaration, this study was allowed by the ethics committee of Nanjing First Hospital (document number: KY20130424-01), and informed consent of all patients was obtained.

### Patient Clinical and Demographic Variables

Data used for prediction were routinely gathered and stored in the electronic health record. Demographic variables included age, education level, and education. Laboratory data included fasting blood glucose (FBG), systolic blood pressure (SBP), and platelet count. The quality of laboratory data was validated throughout the study period by regular internal quality control procedures and participation in an External Quality Assessment scheme. Comorbidities were diagnosed by experienced clinicians and identified according to *International Statistical Classification of Diseases and Related Health Problems, 10th Revision [ICD-10]* codes, including hypertension, diabetes mellitus, and atrial fibrillation. Clinical symptoms included language disorder, facial paralysis, hemiplegia, and dizziness. Medication use history was recorded on admission. Based on the clinical characteristics, imaging, and laboratory examination, ischemic stroke etiology was classified by a trained physician by Trial of Org 10172 in Acute Stroke Treatment (TOAST) criteria (15). NIHSS on admission and 3-month mRS were evaluated by certified assessors during telephone questionnaires or face-to-face interviews with the patients, their relatives, or general practitioners. Data must have been recorded and available in the electronic health record before prediction to be included.

### Statistical Analysis

The continuous variable data was presented as the median value and interquartile range, using Mann-Whitney U test for clinical and demographic comparison between two groups. Univariate tests were conducted using Pearson's chi-square test or Fisher's exact test for categorical data which were indicated as the number of events (fraction of the total). All tests were two-sided and *p*-values < 0.05 were considered statistically significant. The above statistics and descriptions were implemented with SPSS version 25.0 (IBM Corporation, Armonk, NY, USA).

### ML Algorithms

Before introducing the ML prediction model with the demographic and clinical variables mentioned above, missing values were first filled following the k-nearest neighbor algorithm (16). In addition, patients who missed more than one data would be excluded. The continuous data were standardized by z-score normalization (17), and the categorical data were converted by one-hot encoding (18). To select the ML algorithm that exhibits the best predictive ability, five ML classifiers, logistic regression (LR), support vector machine (SVM), random forest classifier (RFC), extreme gradient boosting (XGB), and deep neural network (DNN), were implemented for model construction to predict PSD in mild stroke patients.

### Feature Selection

Superfluous and extraneous factors may lead to model overfitting and affect the predictive power of the model, respectively. Thus, a feature selection process was carried out in the study. All variables with significant difference (*p* < 0.05) in the univariate analysis were subjected to the least absolute selection and shrinkage operator (LASSO) algorithm, which is available for software python (version 3.7; https://www.python.org/). LASSO algorithm implements variable selection and regularization to improve the prediction accuracy and interpretability of the model (19). Finally, variables with non-zero coefficients determined by LASSO method were incorporated for building ML models. The feature selection algorithm was carried out with Python Scikit-learn environment (version 0.23.2).

### Model Development

Supervised ML algorithms mentioned above with binary classification (PSD and non-PSD) were applied to establish predictive models. The study population was randomly divided into the training set (80%) for developing models and the testing set (20%) for assessing the models' performance. In the training step, 10-fold cross-validation was implemented, dividing and generating ten different derivation and inner validation subsets, which improved the generalizability and avoided overfitting. Grid search algorithm was adapted to tune model hyper-parameters to achieve the highest area under curve (AUC) of receiver operating characteristic (ROC).

### Model Evaluation

Upon obtaining the models, the predictive performance was assessed on a testing set according to scores of AUC of ROC, drawn by sensitivity and 1-specificity across a series of cut-off points. Discrimination of the ML model on the testing set was evaluated by AUC. Delong test was carried out to compare the ROC curves in different models. Calibration of the ML model on the testing set was evaluated by calculating Brier score, calibration slope, and calibration intercept. The difference between the estimated and observed risk for PSD was calculated by Brier score, and the model with calibration slope = 1 and calibration intercept = 0 indicated perfect calibration. In addition, the null model Brier score was calculated to compare the relative gain of the algorithms to this benchmark (20). Decision curve analysis was introduced to evaluate the clinical utility (weighted average of true positives and false positives) by calculating the net benefits in the range of threshold probabilities. To evaluate the dominance of the ML models in terms of predictive performance, we also implemented THRIVE and HIAT score on the testing set (10, 11). Finally, the optimal model was selected for DAMS.

### Feature Importance

ML models were accused of being “black boxes,” which means that the development and validation processes of ML models are uninterpretable. In order to rank features in ML models, we introduced the SHapley Additive exPlanations (SHAP) approach. The SHAP approach has a high potential for rationalization of the predictions from sophisticated ML models (21). In addition, the SHAP method indicates whether the effect of a feature on the result is positive or negative.

### Rapid Prediction Model

DAMS may include some variables that take a relatively long time to obtain in emergency contexts, such as triglycerides and creatinine levels. For a more urgent situation, rapid-DAMS (R-DAMS), which excluded these variables, would be constructed based on DAMS. Then we will compare R-DAMS with DAMS in multiple dimensions such as ROC, calibration curve, and decision curve analysis.

## Results

### Study Population

**Figure 1:**
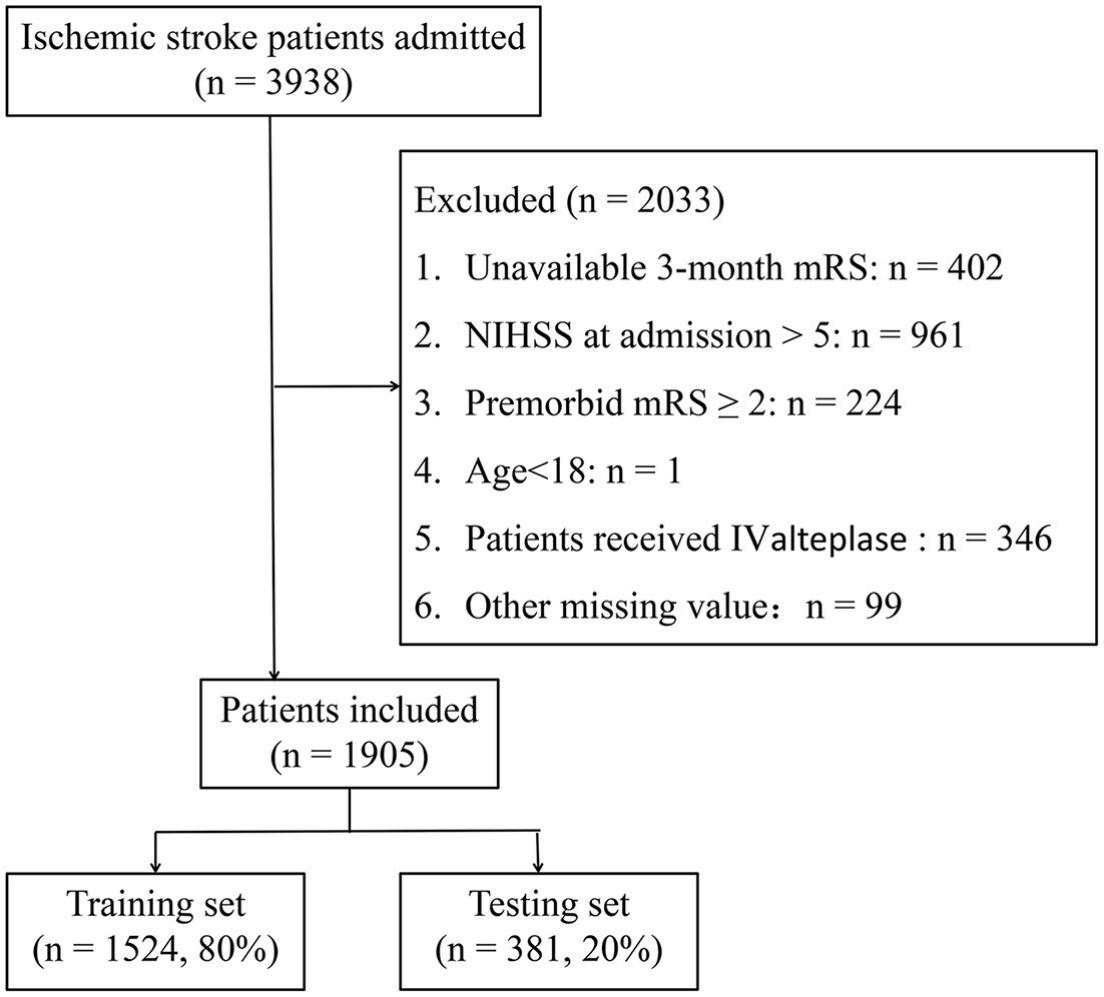
Flow chart illustrating patient selection. mRS, modified Rankin Scale; NIHSS, National Institute of Health stroke scale; IV, intravenous.

As shown in Figure 1, 1,905 patients met the inclusion criteria and were included in the present study. Patients with PSD account for 23.5% (447) of mild stroke patients; analogous proportions of PSD patients were established between training and testing sets (22.9 vs. 25.7%, *p* > 0.05). The median age of included patients was 65 (interquartile range: 58–73) years and 1,337 (70.2%) patients were men. The baseline statistics of both PSD and non-PSD groups were exhibited in Table 1. The characteristics of the patients struck a balance between the training (*n* = 1,524, 80%) and testing (*n* = 381, 20%) sets (Supplementary Table 1).

**Table 1:** Demographic and clinical data of the patients.

	**Total** **(n = 1905)**	**Non-PSD** **(n = 1458)**	**PSD** **(n = 447)**	***p*-value**
**Demographic**				
Age years median (IQR)	65 (58–73)	63 (56–71)	70 (63–79)	<0.001^*^
Male sex n (%)	1337 (70.2%)	1046 (71.7%)	291 (65.1%)	0.007^*^
BMI kg/m median (IQR)	24.57 (22.49–26.67)	24.62 (22.49–26.67)	24.39 (22.22–26.67)	0.089
Education n (%)				0.018^*^
0–6	739 (38.8%)	540 (37.0%)	199 (45.5%)	
6–9	617 (32.4%)	487 (33.4%)	130 (29.1%)	
9–12	392 (20.6%)	314 (21.5%)	78 (17.4%)	
>12	157 (8.2%)	117 (8.0%)	40 (8.9%)	
**Risk factors of n (%)**				
Hypertension	1322 (69.4%)	977 (67.0%)	345 (77.2%)	<0.001^*^
Diabetes mellitus	557 (29.2%)	394 (27.0%)	163 (36.5%)	<0.001^*^
Dyslipidemia	55 (2.9%)	44 (3.0%)	11 (2.5%)	0.538
Coronary artery disease	158 (8.3%)	106 (7.3%)	52 (11.6%)	0.003^*^
Atrial fibrillation	90 (4.7%)	62 (4.3%)	28 (6.3%)	0.079
Previous TIA	8 (0.4%)	7 (0.5%)	1 (0.5%)	0.689
Previous ischemic stroke	196 (10.3%)	136 (9.3%)	60 (13.4%)	0.013^*^
Previous hemorrhagic stroke	46 (2.4%)	27 (1.9%)	19 (4.3%)	0.004^*^
Current smoker	877 (46.0%)	710 (48.7%)	167 (37.4%)	<0.001^*^
Current drink	652 (34.2%)	529 (36.3%)	123 (27.9%)	0.001^*^
**Clinical symptoms** ***n*** **(%)**				
Amaurosis	4 (0.2%)	3 (0.2%)	1 (0.2%)	1.000
Language disorder	61 (3.2%)	44 (3.0%)	17 (3.8%)	0.409
Facial paralysis	840 (44.1%)	612 (42.0%)	228 (51.0%)	0.001^*^
Hemiplegia	1271 (66.7%)	924 (63.4%)	347 (77.6%)	<0.001^*^
Dizziness	225 (11.8%)	176 (12.1%)	49 (11.0%)	0.525
Consciousness disturbance	27 (1.4%)	16 (1.1%)	11 (2.5%)	0.038^*^
Sensory disturbance	293 (15.4%)	230 (15.8%)	63 (14.1%)	0.389
**Medication use history n (%)**				
Previous antiplatelet	206 (10.8%)	144 (9.9%)	62 (13.9%)	0.017^*^
Previous anticoagulation	29 (1.5%)	23 (1.6%)	6 (1.3%)	0.722
Previous statin	115 (6.0%)	84 (5.8%)	31 (6.9%)	0.362
**TOAST classification**				<0.001^*^^†^
LAA (%)	895 (47.0%)	620 (42.5%)	275 (61.5%)	
CE (%)	124 (6.5%)	89 (6.1%)	35 (7.8%)	
SAO (%)	824 (43.3%)	697 (47.8%)	127 (28.4%)	
SOC (%)	16 (0.8%)	13 (0.9%)	3 (0.7%)	
SUC (%)	46 (2.4%)	39 (2.7%)	7 (1.6%)	
**Baseline data**				
Premorbid mRS=1 (%)	90 (4.7%)	52 (3.6%)	38 (8.5%)	<0.001^*^^†^
NIHSS at admission median (IQR)	2 (1–3)	2 (1–3)	3 (2–4)	<0.001^*^^†^
SBP mmHg median (IQR)	143 (130–158)	143 (130–158)	146 (130–160)	0.010^*^^†^
DBP mmHg median (IQR)	85 (80–90)	85 (80–90)	84 (80–90)	0.352
Platelet count 10/L median (IQR)	204 (164–204)	205 (165–241)	201 (158–234)	0.068
Creatinine mmol/L median (IQR)	77 (58–82)	75 (59–81)	82 (58–88)	0.022^*^^†^
FBG mmol/L median (IQR)	5.97 (4.58–6.61)	5.86 (4.55–6.40)	6.31 (4.64–7.36)	0.002^*^^†^
TC mmol/L median (IQR)	4.51 (3.77–5.16)	4.50 (3.77–5.15)	4.54 (3.73–5.19)	0.547
TG mmol/L median (IQR)	1.69 (1.02–1.95)	1.73 (1.04–1.98)	1.58 (0.99–1.80)	0.006^*^^†^
HDL mmol/L median (IQR)	1.08 (0.86–1.19)	1.07 (0.87–1.19)	1.07 (0.86–1.21)	0.917
LDL mmol/L median (IQR)	2.72 (2.12–3.26)	2.70 (2.13–3.24)	2.76 (2.11–3.34)	0.189

*IQR interquartile range; BMI body mass index; TIA transient ischemic attacks; TOAST Trial of Org 10172 in Acute Stroke Treatment; LAA large artery atherosclerosis; CE cardioembolism; SAO small artery occlusion; SOC stroke of other determined cause; SUC stroke of undetermined cause; mRS modified Ranking Scale; NIHSS National Institutes of Health Stroke Scale; SBP systolic blood pressure; DBP diastolic blood pressure; FBG fasting blood glucose; TC total cholesterol; TG triglycerides; HDL high-density lipoprotein; LDL low-density lipoprotein*.

*Data are given as n (%) or median (interquartile range)*.

* *Variables included into the least absolute selection shrinkage operator regression (P<0.05)*.

† *Variables selected by the least absolute selection shrinkage operator regression*.

### Feature Selection

Table 1 shows that 21 features were significantly different (*p* < 0.05) between patients with and without PSD with univariate analyses. Then, nine features without non-zero coefficients were excluded by LASSO regression. The final 12 variables incorporated into ML models were age, NIHSS at admission, SBP, creatinine, FBG, triglyceride, hemiplegia, hypertension, previous ischemic stroke, current drink, premorbid mRS, and TOAST classification.

### Model Performance

**Figure 2:**
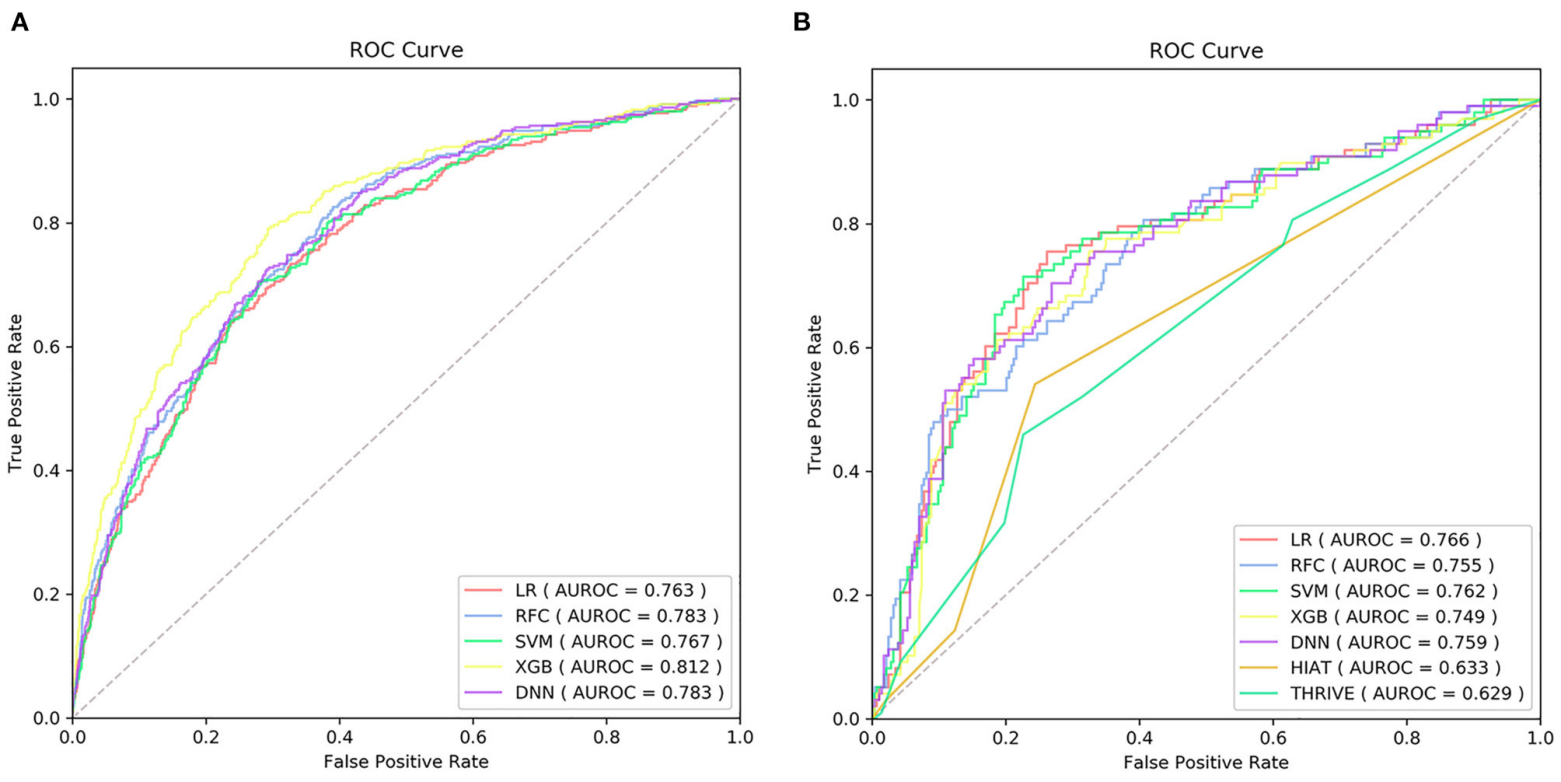
The receiver operating characteristic curve (ROC) of the machine learning models on training set **(A)** and ROC of the machine learning models and traditional models on testing set **(B)** AUC, the area under curve; LR, logistic regression; SVM, support vector machine; RFC, random forest classifier; XGB, extreme gradient boost; DNN, deep neural network; THRIVE, Totaled Health Risks in Vascular Events; HIAT, Houston Intra-arterial Recanalization Therapy.

Supplementary Table 2 exhibited the model hyper-parameters. ROCs of each model on the training set were shown in Figure 2A. Table 2 shows performance metrics on the testing set, including AUC, sensitivity, Brier score, calibration slope, and calibration intercept.

**Table 2:** Discrimination and calibration of each machine learning algorithms on the testing set.

**Model**	**AUC (95% CL)**	**Sensitivity %**	**Specificity %**	**Accuracy %**	**Intercept**	**Slope**	**Brier**
LR	0.766 (0.709–0.823)	78.6	64.3	68.0	−0.129	0.805	0.221
RFC	0.755 (0.699–0.812)	80.6	59.4	64.8	−0.488	1.5533	0.228
SVM	0.762 (0.705–0.819)	74.5	71.0	71.9	0.035	0.935	0.159
XGB	0.749 (0.691–0.807)	70.4	68.2	68.8	0.068	0.879	0.165
DNN	0.759 (0.702–0.816)	74.5	67.1	69.0	−0.030	0.576	0.227

*AUC, area under curve of receiver operating characteristic; CL, confidence interval; LR, logistic regression; RFC, random forest classifier; SVM, support vector machine; XGB, extreme gradient boosting; DNN, deep neural network*.

*Null model Brier score = 0.180*.

As shown in Table 2 and Figure 2B, the discriminative performance was observed in LR (AUC, 0.766; 95% CL, 0.709–0.823), RFC (AUC, 0.755; 95% CL, 0.699–0.812), SVM (AUC, 0.762; 95% CL, 0.705–0.819), XGB (AUC, 0.749; 95% CL, 0.691–0.807), and DNN (AUC, 0.759; 95% CL, 0.702–0.816) on the testing set, and AUCs on the testing set were 0.633 (95% CL, 0.577–0.689) and 0.629 (95% CL, 0.596–0.721) in HIAT and THRIVE score, respectively. The results of the DeLong test indicated that there was no statistical difference in the AUCs of the five ML models, but the AUCs of the five ML models was significantly better than that of HIAT and THRIVE scores (Supplementary Table 3).

**Figure 3:**
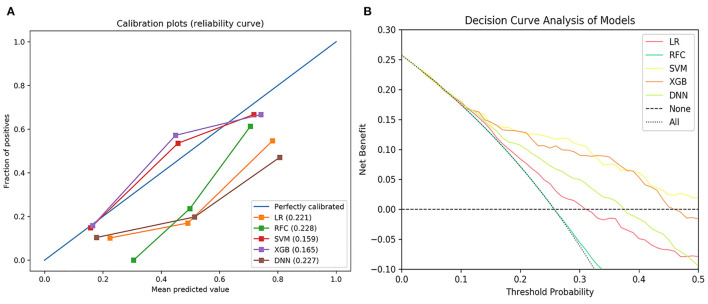
The calibration curve of the machine learning models **(A)** and decision curve analysis of the machine learning models **(B)**. LR, logistic regression; SVM, support vector machine; RFC, random forest classifier; XGB, extreme gradient boost; DNN, deep neural network.

The null model Brier score in the present study was 0.180. On the testing set, the Brier score ranged from 0.159 to 0.228. The calibration slope ranged from 0.576 to 1.553 and calibration intercept ranged from −0.488 to 0.068 (Figure 3A and Table 2). Decision curve analysis indicated that SVM and XGB models exhibited higher net benefit than other ML models as well as default strategies of treating all patients or no patients (Figure 3B).

There was no statistical difference in AUCs of the ML models, but the SVM model exhibited higher net benefit and calibration (Brier score, 0.159, calibration slope, 0.935, calibration intercept, 0.035). Therefore, the SVM model was selected to be DAMS.

### Feature Importance

**Figure 4:**
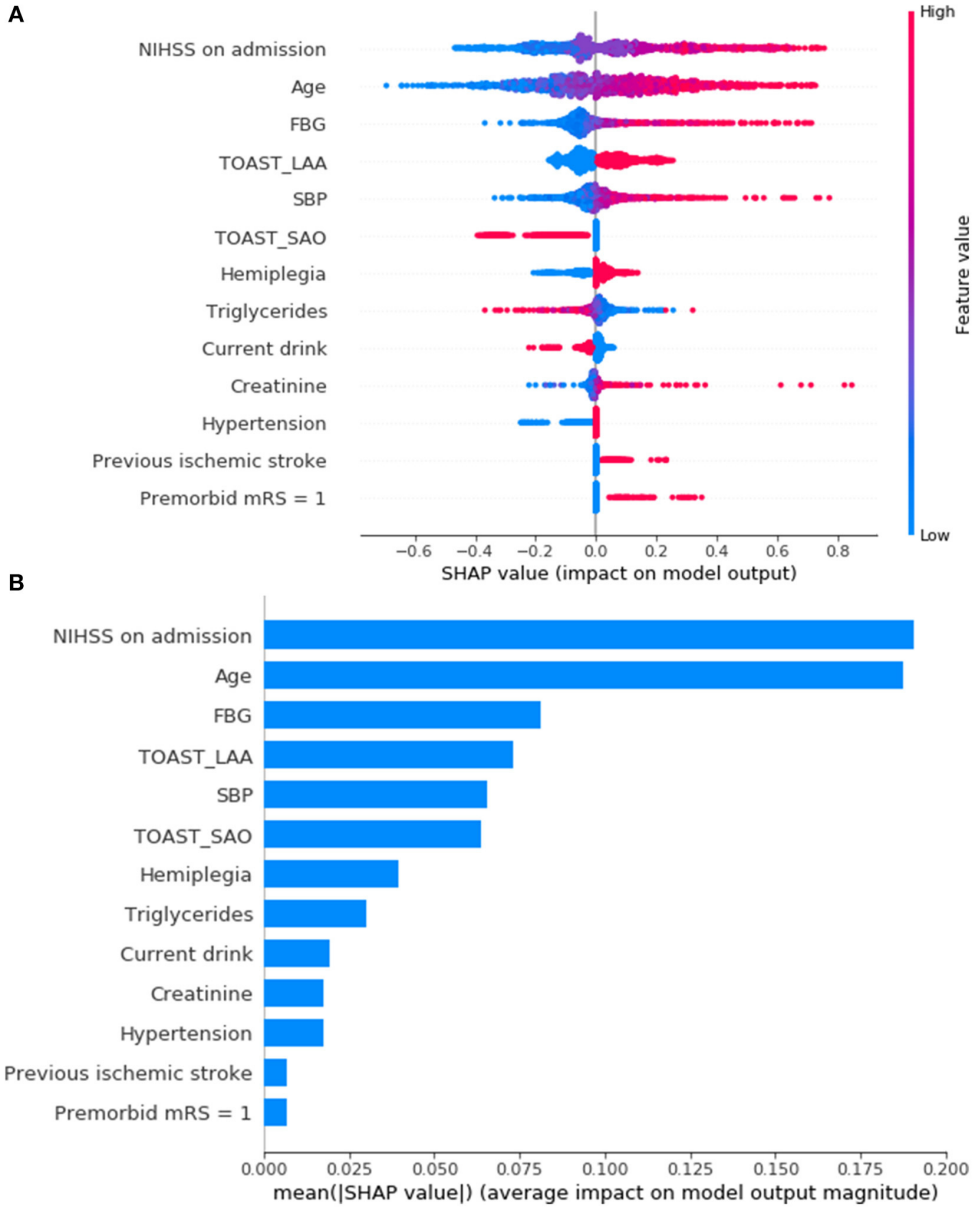
Feature importance ranking based on Shapley Additive exPlanations (SHAP) values **(A,B)** in DAMS. **(A)** Red indicates that the value of the feature is high, and blue indicates that the value of the feature is low; the x-axis represents the SHAP values. The features are ranked according to the sum of the SHAP values for all patients. **(B)** Standard bar charts were drawn and sorted using the average absolute value of the shape values of each feature in DAMS. NIHSS, National Institutes of Health Stroke Scale; FBG, fasting blood glucose; TOAST, Trial of Org 10172 in Acute Stroke Treatment; LAA, large artery atherosclerosis; SAO, small artery occlusion; SBP, systolic blood pressure; mRS, modified Ranking Scale.

SHAP was introduced to rank the feature importance based on DAMS. Figures 4A,B show that the most important features were NIHSS on admission, age, and FBG. Figure 4A shows the individual distribution of SHAP values for single variables on DAMS. The redder the color of the sample dot, the higher the feature value of the variable for the sample. The higher the SHAP value of the abscissa, the greater the likelihood of PSD. Feature importance based on other ML models trained in the present study were provided in Supplementary Figure 1.

### Rapid Prediction Model

**Figure 5:**
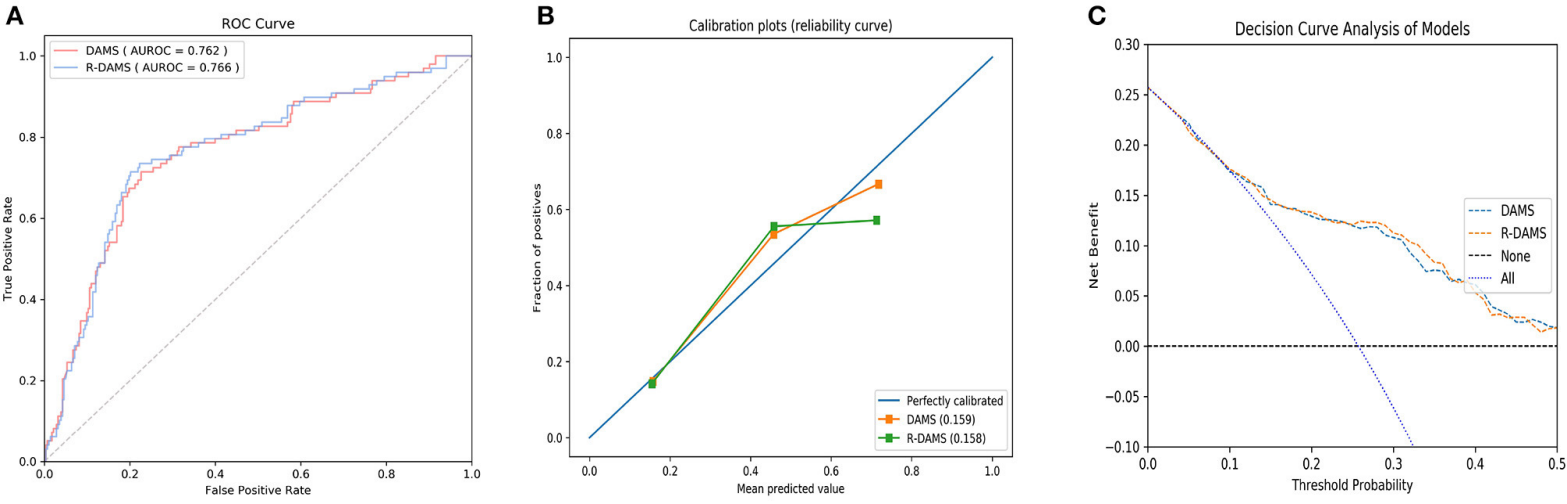
The receiver operating characteristic curve (ROC) **(A)**, the calibration curve **(B)**, and decision curve analysis **(C)** between R-DAMS model and DAMS model. AUC, the area under curve.

DAMS included triglycerides and creatinine levels, which may take some time to obtain in an emergency context. Therefore, rapid-DAMS (R-DAMS) that excluded triglycerides and creatinine levels were constructed for more urgent situations. Then, we compared it with DAMS on a testing set using ROC, calibration curve, and decision curve analysis. As shown in Figure 5 and Supplementary Table 4, there was no significant difference in AUC between R-DAMS and DAMS but the former performed slightly worse on calibration.

## Discussion

In this study, we demonstrated DAMS had the capacity to early identify mild stroke patients who would be at high risk of PSD if they only received medical therapy, achieving an optimal performance compared with our other ML models and previous scoring systems (THRIVE and HIAT scores). In addition, R-DAMS was developed for more urgent situations. DAMS and R-DAMS were able to generate reliable risk estimates for individuals, relying merely on data that were acquired in an emergency setting, and R-DAMS was able to do this within 4.5 h or less of symptom onset. Hitherto none of the prognosis models for mild stroke patients were developed for the prime objective of providing clinical decision support which targets treatment in the emergency contexts. DAMS and R-DAMS, as prediction-driven clinical decision support tools with this target in mind, are significant because neurologists faced a dilemma about the more debatable area of treating mild stroke: using IV alteplase but with the risk of sICH, or not using IV alteplase but potentially leaving the patient with brain ischemia.

In our study, the use of R-DAMS could offer neurologists effective support in the IV alteplase decision. Whether mild stroke patients will benefit from IV alteplase is still controversial. A meta-analysis reported that mild stroke patients who were treated with IV alteplase had lower odds of PSD even if the incidence of sICH increased slightly (22, 23). However, this research relied on retrospective data. The Potential of rtPA for Ischemic Strokes with Mild Symptoms (PRISMS) trial, which prospectively enrolled mild stroke patients without “clearly disabling” deficits, demonstrated no benefit for IV alteplase in this subgroup of patients (23). This trial defined a more certain, but not definitive, population for which the use of IV alteplase cannot be recommended. In line with the findings of the PRISMS trial, the AHA/ASA guidelines distinguish mild disabling stroke from mild non-disabling stroke and recommend IV alteplase within 3 and 4.5 h only for the former (6). The population in our study was not categorized by whether their initial symptoms were “clearly disabling,” because there are subtle differences in judgments about “clearly disabling” deficits in individual neurologists. In the present study, it should be stated explicitly that for patients who were identified to be at high risk of PSD by DAMS or R-DAMS, medical therapy alone is not enough. Thus, the two models support decision-making in the following ways: First, for mild stroke patients judged to be eligible for IV alteplase by current guidelines, R-DAMS was the best choice. The prediction generated by R-DAMS, paired with neurologists' expertise, enables them to choose the most appropriate candidates for IV alteplase. Second, for patients who are not eligible but are at high risk of PSD according to DAMS, best medical therapy alone with close monitoring may be an appropriate course of action.

On the other hand, we unlocked the potential utility of DAMS in secondary prevention. In a secondary analysis of the Acute Stroke or Transient Ischemic Attack Treated with Aspirin or Ticagrelor and Patient Outcomes (SOCRATES) trial, recurrent cerebrovascular event occurred at a significantly higher rate in patients with PSD than patients without PSD (29.0 vs. 3.7%) (5). Furthermore, as a leading cause of PSD (5, 8), a recurrent cerebrovascular event would do more irreparable harm to the patients at high risk of PSD compared with those at low risk. Therefore, effective prevention of recurrent cerebrovascular event to the patients at high risk of PSD portends a decreased risk of PSD. In the present study, DAMS could help to identify mild stroke patients at high risk of PSD, namely those who would most likely obtain substantial benefits from secondary prevention. For this patient group, a focus on evidence-based treatments for secondary prevention, and a support program to improve achievement of secondary prevention targets (e.g., blood pressure, diabetic control, cholesterol) in the long-term, might significantly reduce PSD.

With the expectation that DAMS and R-DAMS can be integrated into clinical practice, we had to acknowledge that our results represent only one step toward one component of a prediction-driven decision support tool for mild stroke patients. Some other steps need to be considered. Firstly, external validation, using data sets from different centres, should be carried out to duplicate the present results. Secondly, an impact study, quantifying whether application of DAMS and R-DAMS in clinical practice improves neurologists' decision making and subsequent patient outcome, is indispensable (24). Finally, development of simple-to-use software, providing a clear interpretation of the prediction and further treatment/prevention information based on this prediction, is required. The present results are promising but we need to emphasize that much work must be done before completely integrating DAMS and R-DAMS into clinical practice.

In the present study, several predictors of PSD have been discovered. NIHSS is a widespread assessment tool used to quantify the baseline severity in stroke patients. As shown in Figure 4A, even within a narrow range of baseline scores, the strongest feature that contributed to the prediction was NIHSS on admission and the higher the values of NIHSS, the more likely the chance of PSD. Noticeably, although the NIHSS has been widely favored in clinical research, some neurological deficits are measured objectively. For example, one NIHSS item, ataxia, confused hemiplegia and normal function by scoring ataxia as “normal” (0) in patients with hemiplegia (25). In the present study, patients with hemiplegia at admission are more likely to be PSD.

There are some limitations to the present study. Firstly, the mRS used to assess the levels of PSD in our study lacks sufficient detail to describe cognition and mood outcomes. A study published in 2017 in the Stroke journal demonstrates that a considerable number of patients with a good mRS outcome were incapable of socially reintegrating because of cognitive impairment and depression (26). However, the validity and reliability of the mRS was recognized by several clinical researchers (23, 27). Since the mRS is easy to use and interpret, the scale has been a valuable tool for assessing the efficacy of therapeutic interventions till now. Secondly, the lack of external validation in our study hinders the evaluation of external generalizability. As a result, whether DAMS and R-DAMS, which have the selection bias that is inherent in any prediction model, can be used directly in other health institutions is still uncertain. To solve this problem, we provided as much detail as possible about the study cohort (Table 1). This information enables other institutions to judge whether their selected population matches the population here. In addition, the process of model development has been described in a precise fashion in Methods and Supplementary Table 2. Therefore, DAMS and R-DAMS may be transferable to other institutions. Thirdly, recurrent cerebrovascular event, a known predictor of PSD in mild stroke patients, was absent in the process by which DAMS and R-DAMS are developed (5, 8). Our models were initially designed for supporting clinical decision-making in emergency contexts, in which the data of recurrent cerebrovascular event is unavailable.

## Conclusions

DAMS and R-DAMS represent one step within a larger process to early identify mild stroke patients who would be at high risk of PSD if they only received medical therapy, by assisting neurologists to make individual clinical decisions for mild stroke patients. Compared with our other ML models and previous scoring systems (THRIVE and HIAT scores), DAMS had a better performance and R-DAMS was able to operate within 4.5 h or less of symptom onset. Future work should build on these findings to transfer DAMS and R-DAMS to different centers.

## Data Availability Statement

The raw data supporting the conclusions of this article will be made available by the authors, without undue reservation.

## Ethics Statement

Written informed consent was obtained from the individual(s) for the publication of any potentially identifiable images or data included in this article.

## Author Contributions

XL formed the conception and study design. XC and JZho did the data collection. NC and FJ did the data analysis. SL and DZ did the literature review and model development. XL and SL drafted the manuscript. ZZ, JZha, and JZo made significant revisions and supplied valuable improvement suggestions. The work presented in this paper was carried out in collaboration with all authors. All authors provided approval of the final version. All authors have read and agreed to the published version of the manuscript.

## Funding

This study received the following financial support: National Natural Science Foundation of China grants 81673511, 81700398, 81970309; and Jiangsu key Research and Development Plan grant BE2017613.

## Conflict of Interest

The authors declare that the research was conducted in the absence of any commercial or financial relationships that could be construed as a potential conflict of interest.

## Publisher's Note

All claims expressed in this article are solely those of the authors and do not necessarily represent those of their affiliated organizations, or those of the publisher, the editors and the reviewers. Any product that may be evaluated in this article, or claim that may be made by its manufacturer, is not guaranteed or endorsed by the publisher.
