# Supplementary material for: Prediction-Driven Decision Support for Patients With Mild Stroke: A Model Based on Machine Learning Algorithms
Source: Front Neurol. 2021 Dec 23;12:761092. doi: 10.3389/fneur.2021.761092 (PMC8733999; doi:10.3389/fneur.2021.761092)
Supplement: Supplementary file 1 [file Data_Sheet_1.docx]

**Supplemental Tables**

**TABLE S1 |** Demographics and clinical characteristics of the training and testing sets

|  | **Training set (n=1524)** | **Testing set (n=381)** | ***p*-value** | % **missing**† |
| --- | --- | --- | --- | --- |
| Demographic | 349’22.9 | 98’25.5 | 0.279 |  |
| Age, years, median (IQR) | 65 (58-73) | 66 (58-74) | 0.479 | / |
| Male sex, n (%) | 1070 (70.2%) | 267 (70.1%) | 0.960 | / |
| BMI, kg/m^2^, median (IQR) | 24.32 (22.49-26.67) | 24.28 (22.60-26.56) | 0.858 | 0.58 |
| Education, n (%) |  |  | 0.858 | 4.25 |
| 0–6 | 594 (39.0%) | 145 (38.1%) |  |  |
| 6–9 | 490 (32.2%) | 127 (33.3%) |  |  |
| 9–12 | 311 (20.4%) | 81 (21.3%) |  |  |
| >12 | 129 (8.5%) | 28 (7.3%) |  |  |
| Risk factors of vessels, n (%) |  |  |  |  |
| Hypertension | 1052 (69.0%) | 270 (70.9%) | 0.486 | / |
| Diabetes mellitus | 421 (27.6%) | 136 (35.7%) | 0.002* | / |
| Dyslipidemia | 884 (58.0%) | 225 (59.1%) | 0.710 | / |
| Coronary artery disease | 121 (7.9%) | 37 (9.7%) | 0.262 | / |
| Atrial fibrillation | 75(4.9%) | 15 (3.9%) | 0.418 | 0.05 |
| Previous TIA | 8 (0.5%) | 0 (0.0%) | 0.330 | / |
| Previous ischemic stroke | 149 (9.8%) | 47 (12.3%) | 0.141 | / |
| Previous hemorrhagic stroke | 40 (2.6%) | 6 (1.6%) | 0.232 | 0.11 |
| Current smoker | 709 (46.5%) | 168 (44.1%) | 0.395 | 0.05 |
| Current drink | 527 (34.6%) | 125 (32.8%) | 0.514 | 0.11 |
| Clinical symptoms, n (%) |  |  |  |  |
| Amaurosis | 2 (0.1%) | 2 (0.5%) | 0.181 | / |
| Language disorder | 45 (3.0%) | 16 (4.2%) | 0.216 | / |
| Facial paralysis | 666 (43.7%) | 174 (45.7%) | 0.489 | / |
| Hemiplegia | 1017 (66.7%) | 254 (66.7%) | 0.981 | / |
| Dizziness | 174 (11.4%) | 51 (13.4%) | 0.287 | / |
| Consciousness disturbance | 16 (1.0%) | 11 (2.9%) | 0.007* | / |
| Sensory disturbance | 226 (14.8%) | 67 (17.6%) | 0.182 | / |
| Medication use history, n (%) |  |  |  |  |
| Previous antiplatelet | 160 (10.5%) | 46 (12.1%) | 0.376 | 0.05 |
| Previous anticoagulation | 25 (1.6%) | 4 (1.0%) | 0.400 | 0.16 |
| Previous statin | 92 (6.0%) | 23 (6.0%) | 1.000 | / |
| TOAST classification |  |  | 0.701 | / |
| LAA (%) | 713 (46.8%) | 182 (47.8%) |  |  |
| CE (%) | 95 (6.2%) | 29 (7.6%) |  |  |
| SAO (%) | 668 (43.8%) | 156 (40.9%) |  |  |
| SOC (%) | 13 (0.9%) | 3 (0.8%) |  |  |
| SUC (%) | 35 (2.3%) | 11 (2.9%) |  |  |
| Baseline data |  |  |  |  |
| Premorbid mRS=1 (%) | 71 (4.7%) | 19 (5.0%) | 0.787 | / |
| NIHSS at admission, median (IQR) | 2 (1-3) | 2 (1-3) | 0.534 | / |
| SBP, mmHg, median (IQR) | 140 (130-158) | 140 (130-160) | 0.797 | 0.21 |
| DBP, mmHg, median (IQR) | 80 (80-90) | 80 (80-90) | 0.881 | 0.11 |
| Platelet count, 10^9^/L, median (IQR) | 198 (164-238) | 198 (164-246) | 0.513 | 1.63 |
| Creatinine, mmol/L, median (IQR) | 69 (59-82) | 71 (57-83) | 0.548 | 0.32 |
| FBG, mmol/L, median (IQR) | 5.17 (4.56-6.58) | 5.24 (4.64-6.85) | 0.133 | 3.01 |
| TC, mmol/L, median (IQR) | 4.43 (3.76-5.15) | 4.39 (3.78-5.17) | 0.741 | 0.21 |
| TG, mmol/L, median (IQR) | 1.39 (1.02-1.95) | 1.42 (1.07-1.93) | 0.384 | 0.11 |
| HDL, mmol/L, median (IQR) | 1.01 (0.87-1.19) | 1.01 (0.85-1.19) | 0.399 | 0.16 |
| LDL, mmol/L, median (IQR) | 2.67 (2.12-3.26) | 2.65 (2.14-3.22) | 0.630 | 0.16 |
| Post-stroke disability | 349 (22.9%) | 98 (25.7%) | 0.245 | / |
| IQR, interquartile range; BMI, body mass index; TIA, transient ischemic attacks; TOAST, Trial of Org 10172 in Acute Stroke Treatment; LAA, large artery atherosclerosis; CE, cardioembolism; SAO, small artery occlusion; SOC, stroke of other determined cause; SUC, stroke of undetermined cause; mRS, modified Ranking Scale; NIHSS, National Institutes of Health Stroke Scale; SBP, systolic blood pressure; DBP, diastolic blood pressure; FBG, fasting blood glucose; TC, total cholesterol; TG, triglycerides; HDL, high-density lipoprotein; LDL, low-density lipoprotein. Data are given as n (%) or median (interquartile range); †The column titled “% missing” represents the percentage of missing values in the total patients. | | | | |

**TABLE S2 |** Hyper-parameters of each model

| Model | Hyper-parameters | parameter values |
| --- | --- | --- |
| LR | C | 1 |
|  | class_weight | 0:1,1:4 |
| RFC | n_estimators | 200 |
|  | max_depth | 3 |
|  | min_samples_split | 50 |
|  | min_samples_leaf | 10 |
|  | class_weight | 0:1,1:4 |
| SVM | C | 0.5 |
|  | gamma | 0.01 |
|  | class_weight | 0:1,1:4 |
| XGB | n_estimators | 20 |
|  | gamma | 0.5 |
|  | max_depth | 2 |
| DNN | Hidden layers | 20-15-4 |
|  | dropout | 0.1 |
|  | class_weight | 0:1,1:4 |
|  | learning rate | 0.2 |
| XGB indicates extreme gradient boosting; LR, logistic regression; RFC, random forest classifier; SVM, support vector machine; DNN, deep neural network. | | |

**TABLE S3 |** The *p*-values of pairwise comparisons of AUCs on testing set for different models with Delong test

| Model | RFC | SVM | XGB | DNN | THRIVE | HIAT |
| --- | --- | --- | --- | --- | --- | --- |
| LR | 0.4044 | 0.3496 | 0.1923 | 0.5209 | 0.0001 | <0.0001 |
| RFC |  | 0.5438 | 0.5373 | 0.7612 | 0.0007 | 0.0001 |
| SVM |  |  | 0.2647 | 0.7588 | 0.0002 | <0.0001 |
| XGB |  |  |  | 0.4390 | 0.0013 | 0.0002 |
| DNN |  |  |  |  | 0.0004 | 0.0001 |
| THRIVE |  |  |  |  |  | 0.9241 |
| The significant difference between AUCs is defined as *p*-value < 0.05.  Abbreviations: AUC, the area under receiver operating characteristic curve; XGB indicates extreme gradient boosting; LR, logistic regression; RFC, random forest classifier; SVM, support vector machine; DNN, deep neural network; THRIVE, Totaled Health Risks in Vascular Events; HIAT, Houston Intra-arterial Recanalization Therapy. | | | | | | |

**TABLE S4 |** Discrimination and calibration between DAMS and R-DAMS on the testing set

| Model | AUC (95% CL) | Sensitivity | Specificity | Accuracy | Intercept | Slope | Brier |
| --- | --- | --- | --- | --- | --- | --- | --- |
| DAMS | 0.762 (0.705-0.819) | 74.5 % | 71.0 % | 71.9 % | 0.035 | 0.935 | 0.159 |
| R-DAMS | 0.766 (0.691-0.807) | 77.6% | 65.4% | 68.5% | 0.074 | 0.789 | 0.158 |
| AUC, area under curve of receiver operating characteristic; CL, confidence interval; LR, logistic regression. Null model Brier score=0.180. | | | | | | | |

**Supplemental Figures**


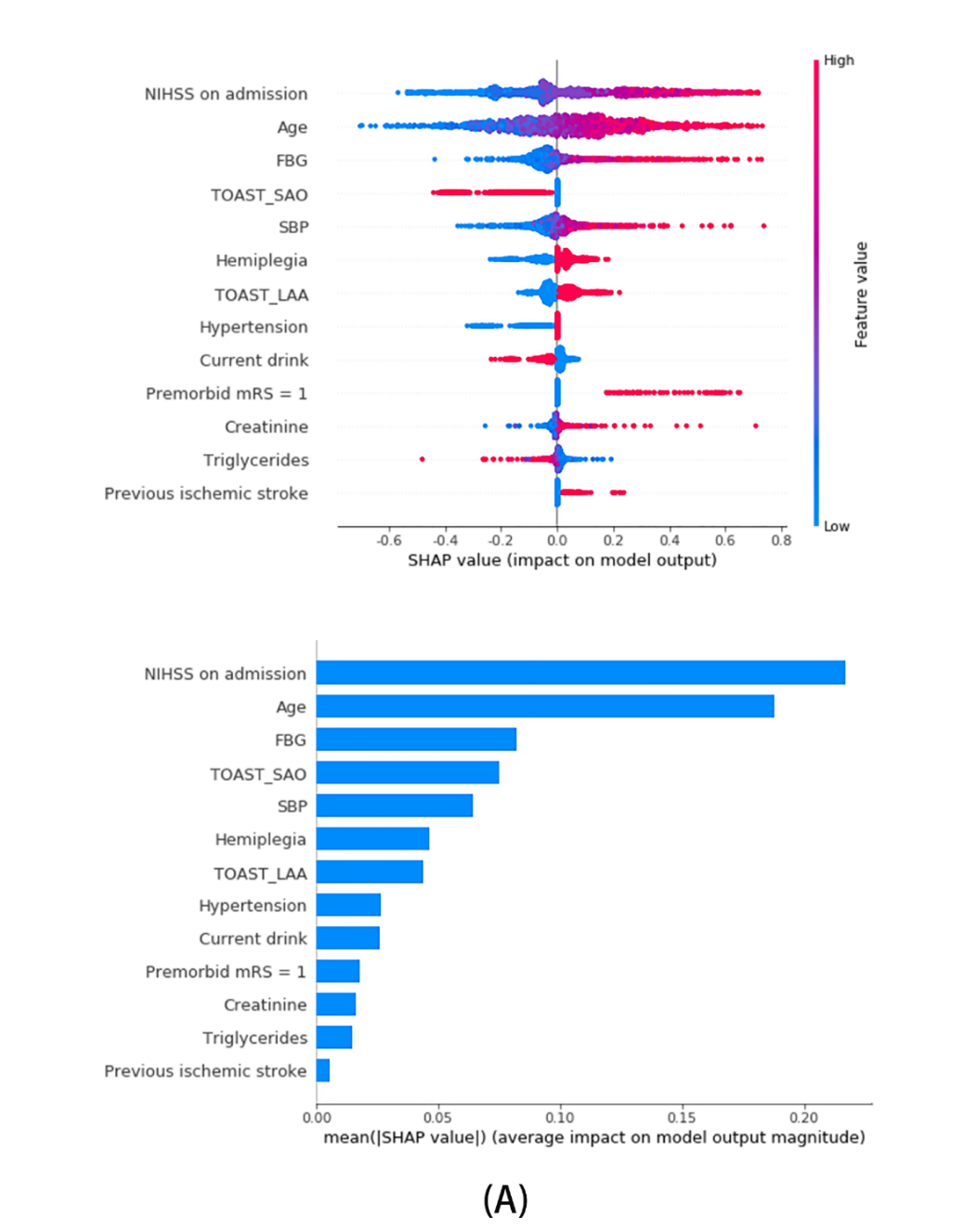


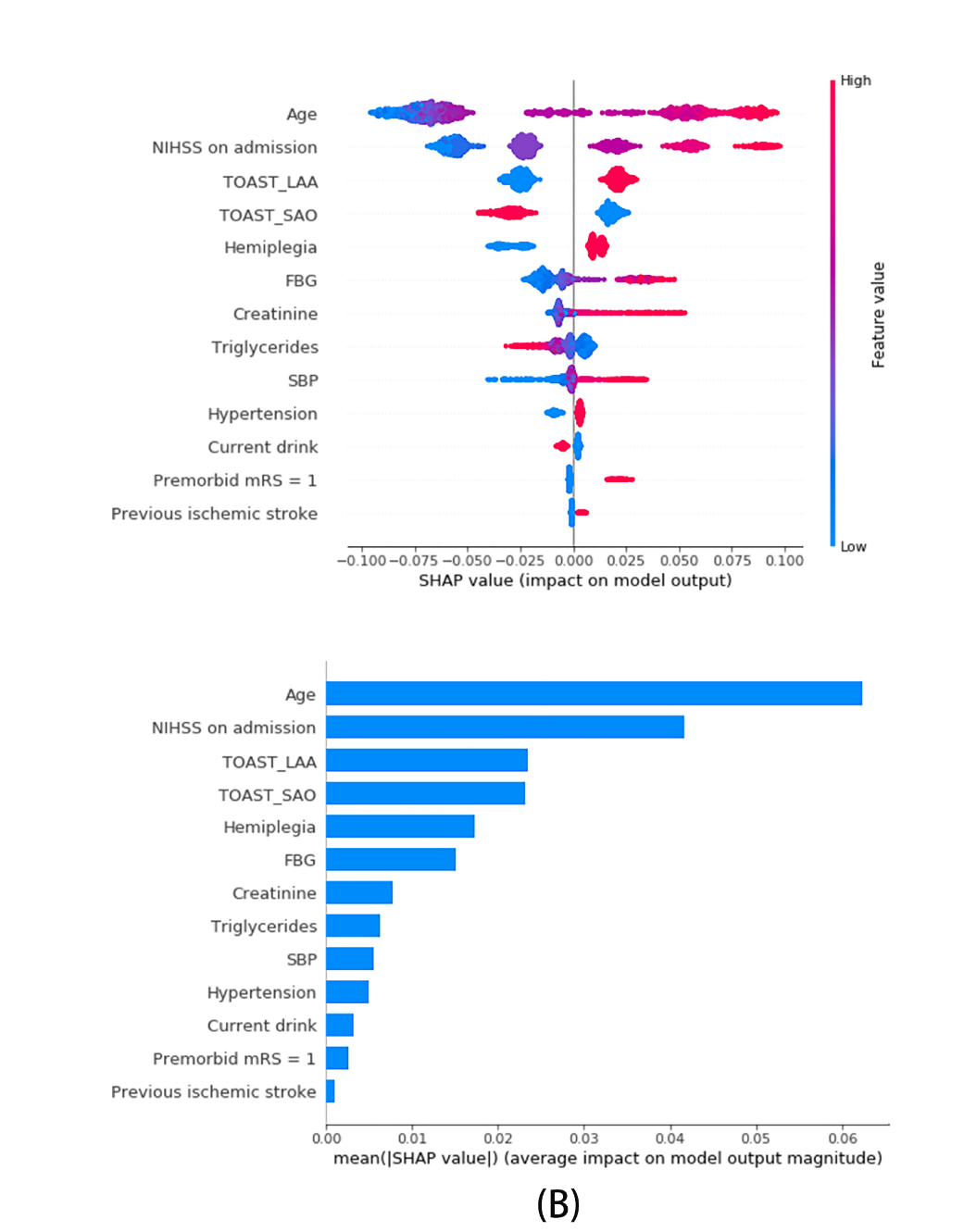


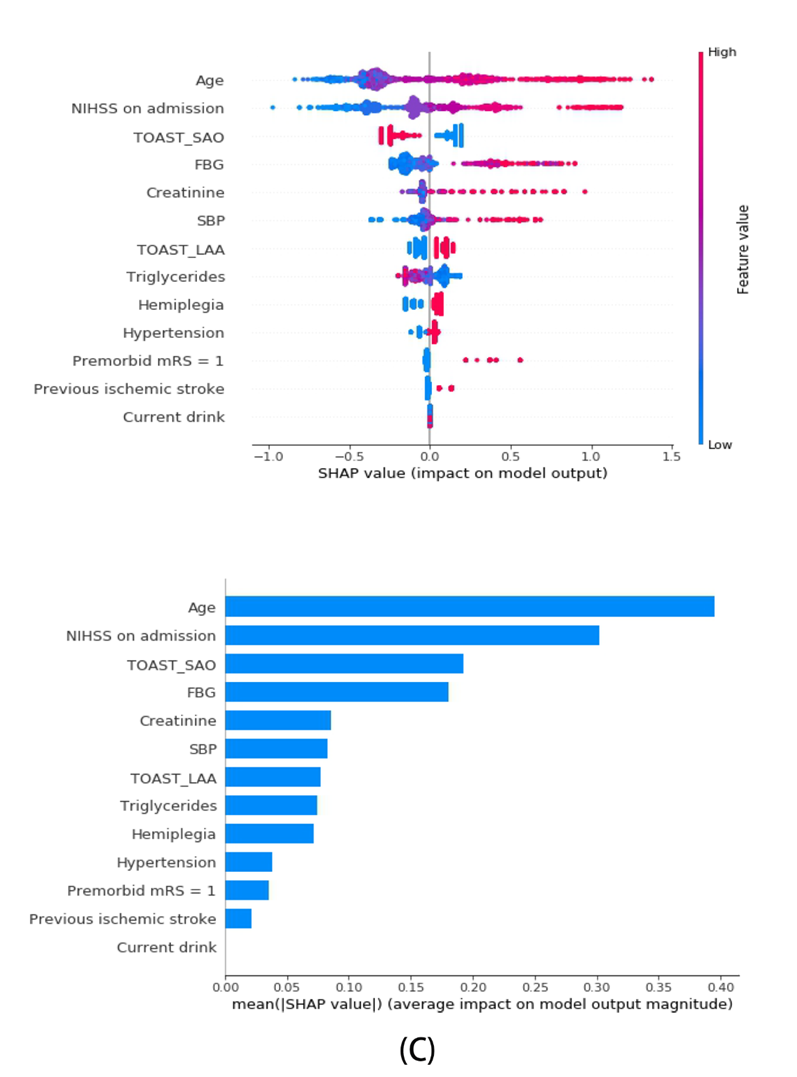


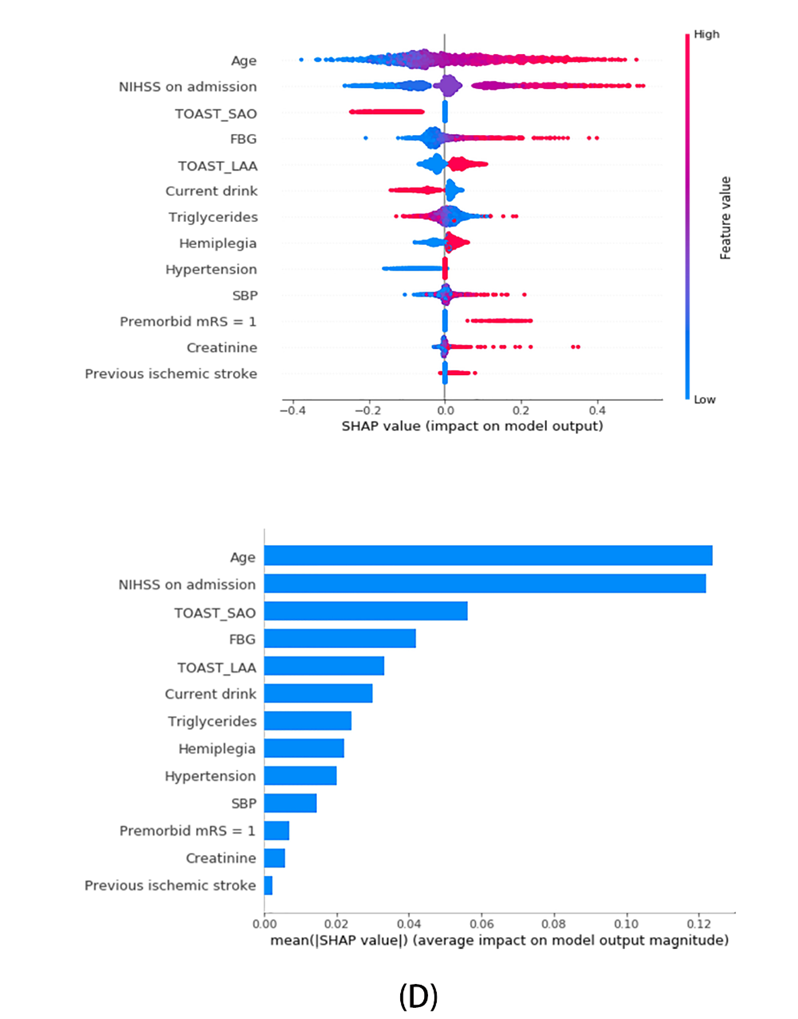


**FIGURE S1 |** Feature importance ranking based on SHapley Additive exPlanations (SHAP) values in (A) logistic regression, (B) random forest classifier, (C) extreme gradient boost, (D) deep neural network.
